# Mapping the interaction between Trim28 and the KRAB domain at the center of Trim28 silencing of endogenous retroviruses

**DOI:** 10.1002/pro.4436

**Published:** 2022-09-21

**Authors:** Jamie R. H. Taka, Yunyuan Sun, David C. Goldstone

**Affiliations:** ^1^ School of Biological Sciences University of Auckland Auckland New Zealand; ^2^ Maurice Wilkins Centre for Molecular Biodiscovery Auckland New Zealand

**Keywords:** Alphafold2, binding interface, Kap1, KRAB domain, protein–protein interaction, Trim28

## Abstract

Transcription of endogenous retroviral elements are tightly regulated during development by members of the KRAB‐containing zinc finger proteins (KRAB‐ZFPs) and the co‐repressor Trim28 (also known as Kap‐1 or Tif1β). KRAB‐ZFPs form the largest family of transcription regulators in mammals and initiate transcriptional silencing by tethering Trim28 to a target locus. Subsequently, Trim28 recruits chromatin modifying effectors resulting in the formation of heterochromatin. In the present study, we identify surface exposed residues on the central six turns of the Trim28 coiled‐coil region forming the binding interface for the KRAB domain. Using AlphaFold2 (AF2) we provide high confidence models of the interface between Trim28 and the KRAB domain and identified leucine 301 on each chain of the Trim28 monomer to act as a pin extending into a hydrophobic pocket on the KRAB domain surface. Site directed mutations in the Trim28‐KRAB binding interface abolished binding to the KRAB domain. Our work provides a detailed understanding of the specific interactions between the KRAB domain and the Trim28 coiled‐coil and how this interaction may be regulated during silencing events.

**Abbreviations**
KRAB‐ZFPs: KRAB‐containing zinc finger proteins

## 1 INTRODUCTION

The human genome contains endogenous retroviral elements (ERVs) as relics of historic retroviral infections that have been maintained in the germline and inherited by future generations. In human ancestral lineages it is estimated there were over 50 waves of retroviral endogenization events.^1^ These ERVs can retain the ability to amplify their genetic material and reintegrate into the host genome, resulting in retroviral‐like sequences comprising approximately 8% of the human genome.^1^ Unregulated insertion of retrotransposons can result in genomic instability, thus ERV's are targeted for repression. Members of the KRAB‐containing zinc finger proteins (KRAB‐ZFPs) and the co‐repressor Tripartite motif 28 (Trim28) target ERVs during embryonic development to prevent expression.^2^ Targeted repression of ERVs is also recognized as an essential contributor to gene regulatory networks during development and in differentiated cells.^3^, ^4^, ^5^

The KRAB‐ZFPs contain an N‐terminal KRAB domain followed by an array of between three and 40 DNA binding C2H2 zinc finger domains.^6^ The zinc‐finger domains provide sequence specificity, recognizing three consecutive nucleotides on the primary DNA strand and one nucleotide on the secondary DNA strand. The KRAB domain consists of a ~72 amino acids arranged as KRAB‐A and KRAB‐B subdomains. The KRAB‐A domain is required and is sufficient for Trim28 binding and to induce repression.^7^, ^8^, ^9^ The KRAB‐B subdomain can enhance repression by an undetermined mechanism.^8^, ^10^ KRAB‐ZFPs have undergone rapid expansion in mammalian genomes with this expansion correlated with the appearance of new families of ERVs.^11^ In humans there are 381 genes, generating over 700 proteins.^12^, ^13^ The principal function of KRAB‐ZFPs is to recruit the universal co‐repressor Trim28 to specific sites in the genome via the KRAB‐A domain, where Trim28 assembles a macromolecular complex containing chromatin remodeling proteins including SWI/SNF‐Related, Matrix‐Associated Actin‐Dependent Regulator Of Chromatin, Subfamily A, Containing DEAD/H Box 1 (SMARCAD1), SET domain bifurcated 1 (SETDB1), nucleosome remodeling and histone deacetylation (NuRD) complex, and Heterochromatin protein 1 (HP1).

Trim28 (or KAP1 or TIF1β) belongs to the TRIM protein family of RING E3 ubiquitin ligases consisting of over 70 members in humans. Family member's share a conserved N‐terminal domain organization termed the RBCC, consisting of a RING finger domain, one or two B‐box domains, and a coiled‐coil region. One defining feature of TRIM proteins is their ability to oligomerize as homo‐dimers and higher order oligomers. The coiled‐coil regions of TRIM proteins forms an extended ~170 Å long α‐helix that exhibits a conserved heptad repeat forming a constitutive anti‐parallel homodimer.^14^, ^15^, ^16^, ^17^, ^18^ This architecture maintains the RING and Bbox domains at either end of the molecule. The RING domain confers E3 ubiquitin ligase activity and commonly requires oligomerization for enhanced activity.^19^, ^20^ The C‐terminal domains vary between family members and are often involved in protein–protein interactions.^21^ Trim28 belongs to the TIF1 subfamily and contains a C‐terminal PHD‐Bromo domain. SUMOylation of the PHD‐Bromo facilitates recruitment of the chromatin remodeling SETDB1 methyltransferase and the NURD deacetylase complex and is required for Trim28's repressive activity.^22^, ^23^, ^24^ Trim28 also directly recruits HP1 by the canonical HP1‐binding motif PXVXL, that in turn binds the repressive H3K9Me3 histone modification and is essential for gene silencing.^25^, ^26^

Two recent *in vitro* investigations on Trim28 binding to the KRAB domain of KRAB‐ZFPs independently determined that the binding stoichiometry is 1:2 KRAB‐ZFP: Trim28, indicating the KRAB binds at the two fold symmetry axis of the coiled‐coil domain.^27^, ^28^ A low resolution small angle x‐ray scattering model of the Trim28 tripartite motif bound to an MBP‐ZFP809 KRAB fusion protein shows Trim28 forms an elongated dumbbell shape dimer with a centrally bound MBP‐KRAB.^28^ Furthermore, mutagenesis of surface exposed residues located at the center of the coiled‐coil, specifically V294 and K297 moderately reduce or abrogate binding.^27^, ^28^

Protein structure prediction from the amino acid sequence has been a developing field for the past decades with the CASP14 experiment^29^ showcasing the latest leap in the field of protein structure prediction with the use of neural network architectures. AlphaFold2 (AF2) developed by Google Deepmind is able to generate highly accurate models given an informative multiple sequence alignment.^30^ Additionally, AF2 can be exploited for its ability to predict cross‐chain contacts in homomers and hetero‐complexes, successfully predicting heteromeric interfaces.^30^, ^31^

In this study we demonstrate the surface exposed residues of the central six turns of the Trim28 coiled‐coil region form the binding interface for the KRAB domain. We provide high confidence models of the interface between Trim28 and the KRAB domain and identified leucine 301 on each chain of the Trim28 monomer to act as a pin extending into a hydrophobic pocket on the KRAB domain surface. We also validate the proposed model through site‐directed mutagenesis of interfacing residues on the Trim28 coiled‐coil that result in a reduction in binding affinity.

## 2 RESULTS

### 2.1 KRAB‐ZFPs bind the center of the coiled‐coil domain of Trim28

**FIGURE 1:**
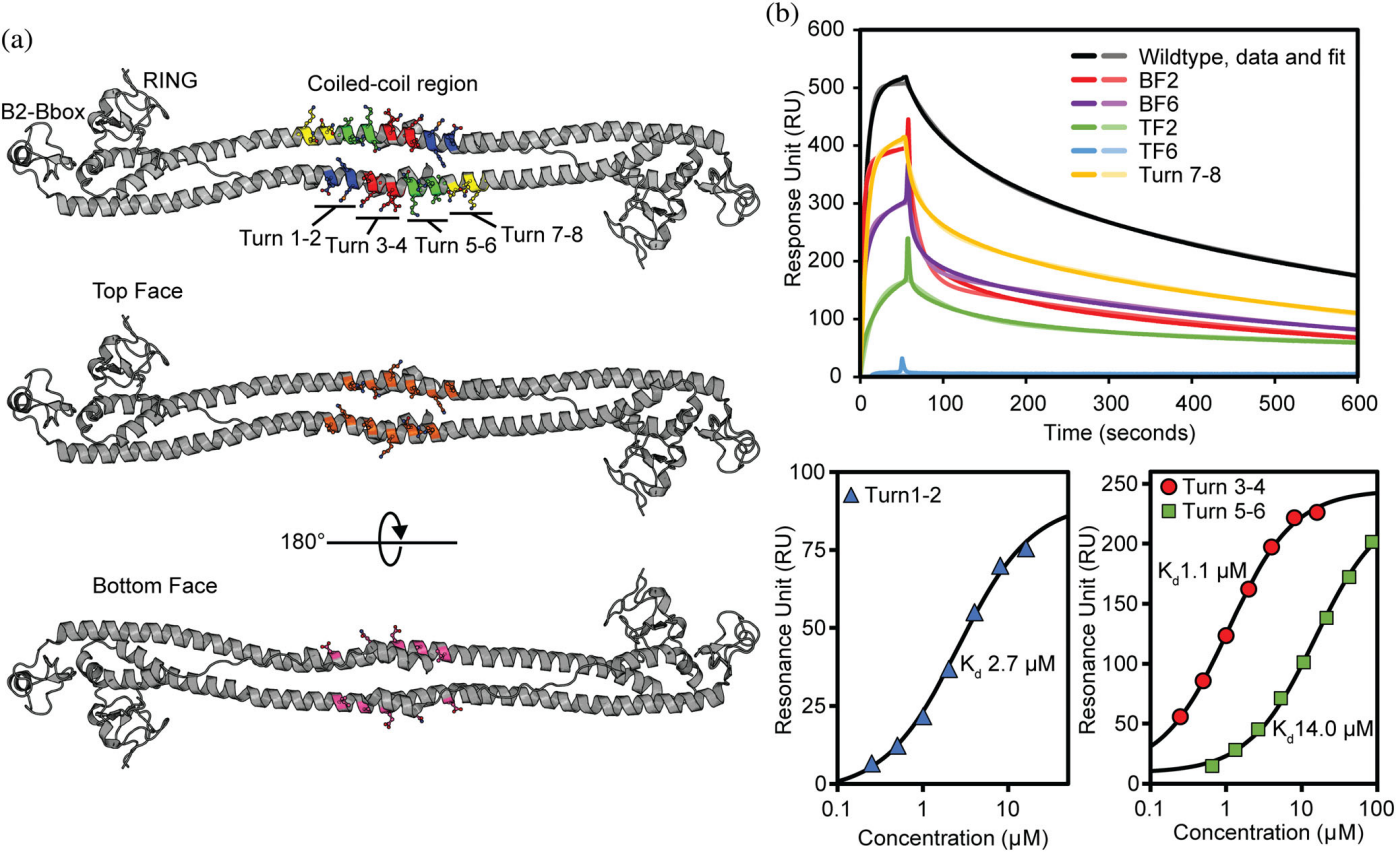
SPR confirms the KRAB domain binds the central region of the coiled‐coil. (a) Cartoon representation Trim28's RBCC crystal structure (PDB ID: 6QU1) with the turn and face mutant residues shown as sticks. Turns 1–2 (blue), Turns 3–4 (red), Turns 5–6 (green), Turns 7–8 (yellow), Top Face (orange), and Bottom Face (purple). (b) Biacore SPR kinetic and equilibrium analysis of Trim28 coiled‐coil mutants over the surface of a CAP sensor chip immobilized with biotinylated Krab domain of ZFP932

The coiled‐coil region of Trim28 is comprised of two 30‐turn amphipathic helices followed by a hairpin loop with the protein chain extending back toward the center of the helices and connecting to a small helix forming a four‐helix bundle.^15^ We have previously mapped the interaction between Trim28 and members of the KRAB‐ZFP family to the center of the Trim28 coiled coil using a set of rationally designed mutations covering the central area of the coiled coil.^28^ These mutants focused on adjacent turns of the coiled‐coil, targeting the central eight turns between residues V294‐V321 of the helices Turns 1–2 (V294A, D295A, K297A, M298A), Turns 3–4 (L301A, Q302A, I303A, K305A, E306A), Turns 5–6 (N308A, K309A, R310A, R312A, V313A, L314A), Turns 7–8 (N316A, D317A, Q319A, K320A, V321A), and on the two opposite “faces” of the coiled coil the top‐face (V294A, K297A, L301A, K305A, N308A, R312A) and bottom‐face (D295A, Q302A, E306A, R310A, L314A) (Figure 1 and Table 1).

**TABLE 1:** Summary of residues targeted for mutation in Trim28 coiled‐coil and the affinity to ZFP932 KRAB domain

Mutant name	Mutations in MuTrim28	Dissociation constant (nM)	Fold‐decrease
Wild type		14.6 ± 1.1	
Turns 1–2	V294A, D295A, K297A, M298A	2.7 ± 0.1 μM	185
Turns 3‐4	L301A, Q302A, I303A, K305A, E306A	1.1 ± 0.1 μM	77
Turns 5–6	N308A, K309A, R310A, R312A, V313A, L314A	14.0 ± 0.7 μM	962
Turns 7–8	N316A, D317A, Q319A, K320A, V321A	61.5 ± 4.0	4
Top Face 2	K305A, N308A	613.4 ± 127.7	42
Top Face 6	V294A, K297A, L301A, K305A, N308A, R312A	Binding deficient	**—**
Bottom Face 2	E306A, R310A	34.5 ± 11.3	2
Bottom Face 6	D295A, Q302A, E306A, R310A, L314A	52.9 ± 7.8	4

*Note*: Residues of the central eight turns of the MuTrim28 coiled‐coil region were mutated to alanine. The binding strength of the Trim28 coiled‐coil mutants to the ZFP932 KRAB domain were assessed by SPR. The data are presented as the average of three equivalent runs plus/minus the standard deviation. The affinities are presented in nM, unless otherwise specified.

Using a series of pull‐down experiments we identified K297 in this central region as being critical for binding. To expand on this work we hypothesized that there are likely other mutations that, rather than abolishing binding completely, would result in a reduction in binding affinity that could be assessed using our surface plasmon resonance (SPR) binding assay.

We undertook Biacore SPR on the Trim28 mutants to examine their binding to the immobilized KRAB domain from ZFP932. The KRAB domain was expressed with an N‐terminal Avitag resulting in a monobiotinylated construct that was then immobilized onto the surface of a biacore Sensor Chip CAP. Data were analyzed by either equilibrium or kinetic analysis.

The interaction between Trim28 and ZFP932 has an affinity of 14.6 ± 1.1 nM consistent with our previous experiments. Analysis of the Turns 1–2 mutant binding to ZFP932 has an affinity of 2.7 ± 0.1 μM, a 185‐fold decrease in affinity. Additionally, K297 was located in the Top Face 6 mutant and was binding‐deficient suggesting that this is the key binding interface. This is supported by mutations on the opposing face of the coiled coil that showed minimal effect on binding with affinities of 34.5 ± 11.3 nM and 52.9 ± 7.8 μM for the bottom face 2/6 mutants, respectively. We were unable to use a kinetic analysis for Turns 3–4 and Turns 5–6 due to the fast on/off kinetics of binding. Equilibrium analysis of Turns 3–4 gave an affinity of 1.1 ± 0.1 μM and Turns 5–6 gave an affinity of 14.0 ± 0.7 μM showing mutations in these turns of the coiled‐coil can disrupt binding to ZFP932 as severe as the K297 mutations. The Turns 7–8 mutant displayed the same affinity as the wild‐type protein placing this area of the coiled‐coil outside the binding interface. Taken together, our binding data confirms that the KRAB domain binds the top face of the Trim28 coiled‐coil and spans the central region including residues between V293 and R311. Furthermore, it demonstrates that residues located on the top face of the Turns 1–6 are involved in the interaction expanding the binding interface compared to previous studies^27^, ^28^ while residues located on Turns 7–8 are not involved.

### 2.2 A model of the KRAB‐Trim28 interface from Alphafold

Recent advances in protein folding algorithms have made it possible to model proteins with a high degree of accuracy. Alphafold has been shown to accurately predict the structure of proteins from sequence alone. Based on the conserved nature of the Trim28‐KRAB interaction, we hypothesized that sufficient co‐evolutionary information would be present for Alphafold2 to provide an accurate model of the interaction that could then be verified either by site‐directed mutagenesis or mutations present in existing literature.

**FIGURE 2:**
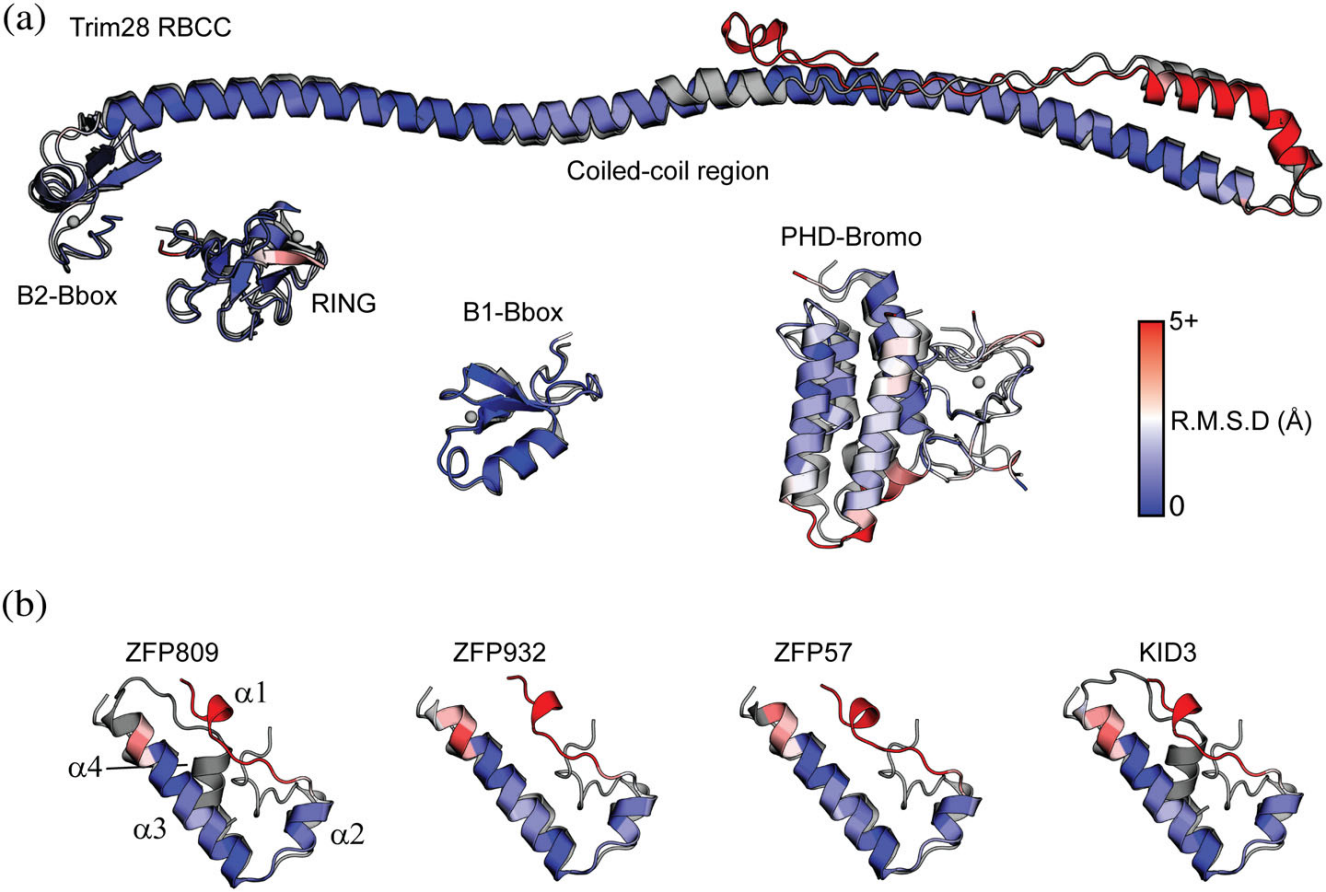
Comparison of AlphaFold models with experimentally determined structures. (a) The Trim28 AlphaFold model is similar to the experimental structures (gray) for the RBCC (PDBid 6QU1), B1‐Bbox (PDBid 6O5K), and the PHD‐Bromo (PDBid 2RO1). (b) The AlphaFold models (blue) of ZFP809, ZFP932, ZFP57, and KID3 resemble the solution structure of a mouse KRAB domain (gray, PDBid 1V65). The AlphaFold model is colored blue through red based on an all atom RMSD comparison to experimentally determined structure

Models for individual protein subunits were accessed via the EBI AlphaFold 2 database. The AF2 predicted structure for Trim28 domains is consistent with published structures (UniProt Q13263) albeit as a model of the monomer rather than the dimer (Figure 2a). However, comparison of the model to the published coiled‐coil structure recreates the monomer structure accurately with the dimer easily generated by superposition of two copies on the published structure. The RING and the B2 Bbox domains are placed in agreement with the published model at one end of the ~170 Å long helix of the coiled‐coil region. The B1 Bbox domain is connected by low confidence linkers indicating the position relative to the other domains is variable. Uncertainty in the position of the B1 Bbox domain is consistent with the published structures that have no interpretable electron density for the B1 Bbox domains when present.^15^, ^27^ The predicted N‐terminal RING, B1 Bbox, and B2 Bbox domains are highly similar to the published structures of individual domains (PDB entries 6QU1 and 6O5K), with r.m.s.d. values of 1.963, 1.447, and 1.741 Å, respectively when comparing all equivalent atoms. In addition to agreeing with the published structures, the residue‐level confidence estimates pLDDT (Local Distance Difference Test) within the N‐terminal domains indicate a highly confident model prediction with the B2 Bbox and coiled‐coil region having scores exceeding 90 pLDDT.

Models of the KRAB‐ZFP KRAB domain from multiple models in the AF2 database converge on a common fold consisting of four helices (Figure 2b). The N‐terminus is an extended coil that extends the length of the domain with a single turn of helix in the middle. The second helix is approximately two turns in length and is immediately followed by a sharp turn and the major helix α3. This helix is 20 amino acids in length and is the major secondary structure element in the domain. A short turn extends into helix 4 oriented at approximately 30° to helix α3. The N‐terminal extended region is positioned between helix α3 and α4. Helices α1‐3 correspond to the conserved KRAB‐A subdomain and are found in all KRAB domains, whereas α4 is only found in KRAB‐ZFP's with a KRAB‐B subdomain. The KRAB domain is predicted with confidence scores between 70 and 90 pLDDT across the four helices indicating an intermediate level of confidence, however AF2 consistently produces a common fold. The C‐terminus of KRAB‐ZFP's typically extends to an array of Zinc‐finger domains that are responsible for DNA recognition. This model is in good agreement with the NMR model of the KRAB domain from uncharacterized protein LOC72139 (PDBid 1V65). The NMR model consists only of helix α2‐α3 and has an r.m.s.d. of 2.563 Å over all equivalent atoms with the KRAB domain from the AF2 model for ZFP809.

To understand the molecular determinants for binding of the KRAB domain with Trim28 we modeled Trim28 in complex with the KRAB domain from ZFP809 using AF2. This modeling is complicated by the 2:1 stoichiometry of the TRIM‐KRAB complex. To generate the model we initially used a sequence whereby the Trim28 RBCC (58–418) was duplicated with a 40‐glycine linker placed between monomers, the KRAB‐AB domain of ZFP809 (residues 1–74) was then appended to the sequence after another 40aa glycine linker to generate the appropriate 2:1 stoichiometry of the interaction.^28^ With advances in the AF2 algorithm we transitioned to using a sequence consisting of the Trim28 RBCC (residues 57–418) with an oligomeric state of two, and a separate chain of the KRAB domain with an oligomeric state of one. Both sequences resulted in equivalent output models. No templates were included in the modeling and the models were relaxed using Amber‐Relax as part of the AF2 modeling process.

**FIGURE 3:**
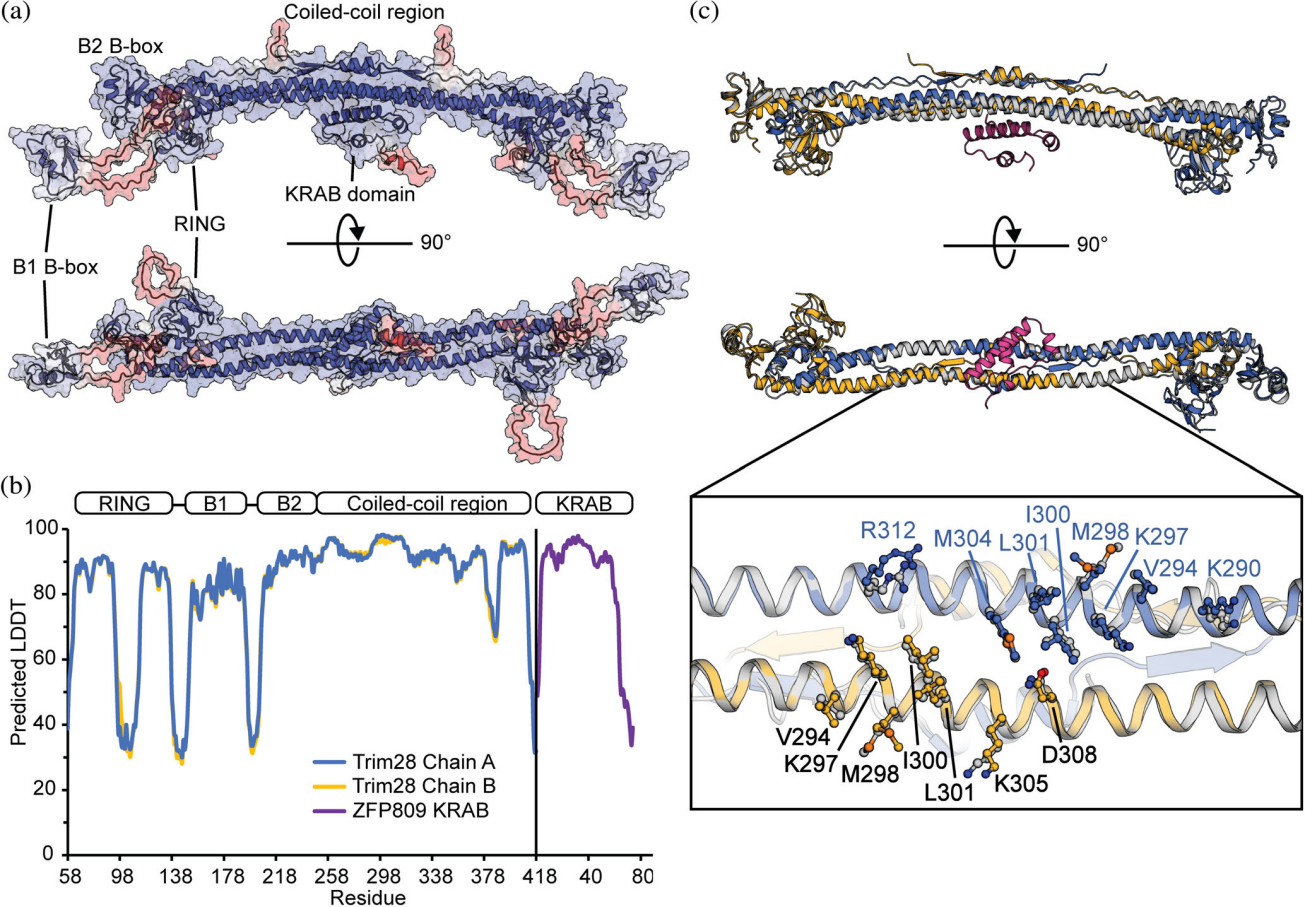
AF2 model of the Trim28 RBBC in complex with the ZFP809 KRAB domain. (a) Surface and cartoon representation. Residues are colored red‐to‐white‐to‐blue for pLDDT estimates ranging from 50 to 100, respectively. (b) A graph of the predicted LDDT for each residue of each chain of Trim28 and the KRAB domain. (c) A structural alignment of the modeled complex with the experimental model for the RBCC (PDBid 6QU1). Residues at the center of the coiled‐coil domain and interfacing with the KRAB domain are shown as ball and sticks

The model for the complex of Trim28 with the ZFP809 KRAB domain comprised of two similar chains of the Trim28 RBCC (r.m.s.d of 3.5 Å for all equivalent atoms) and a single KRAB domain. AF2 recreated the expected anti‐parallel Trim28 dimer with the Bbox and RING domains placed in positions consistent with the published structures (Figure 3a). In our model the KRAB domain is positioned at the center of the coiled‐coil region and shows no interactions with the RING, B1 Bbox, and B2 Bbox domains consistent with experimental mapping of the KRAB binding site.^27^, ^28^ Each domain within the RBCC and the KRAB domain were predicted with high residue‐level confidence estimates ranging from 80 to 92 pLDDT within the RING and B1 Bbox domains, and pLDDT > 90 within the B2 Bbox, coiled‐coil region, and KRAB domain (Figure 3b). Residues within the linkers connecting to the B1 Bbox and the extended loop of the RING domain are modeled with low confidence, indicating variability in their position that is in agreement with known structural data.^15^, ^32^ The model for Trim28 in our complex is highly similar to the published structure for Trim28 RBCC with an r.m.s.d of 2.7 Å for all equivalent atoms, including sidechain conformations at the center of the coiled‐coil region that interface with the KRAB domain (Figure 3c). Furthermore, the predicted structure of the ZFP809 KRAB domain in complex with Trim28 is consistent with the modeled KRAB domain of ZFP809 from the Alphafold database (UniProt G3X9G7) with an r.m.s.d of 0.93 Å for all equivalent atoms. Importantly, residues of the KRAB domain are modeled with equivalent confidence estimates as the interfacing coiled‐coil region of Trim28, and the predicted aligned error (PAE) show a strong correlation between the KRAB domain and central coiled‐coil region of the Trim28 dimer consistent with the correct positioning of this domain (Figure S1). Taken together, the confidence outputs indicate a highly confident model with appropriate domain positions.

**FIGURE 4:**
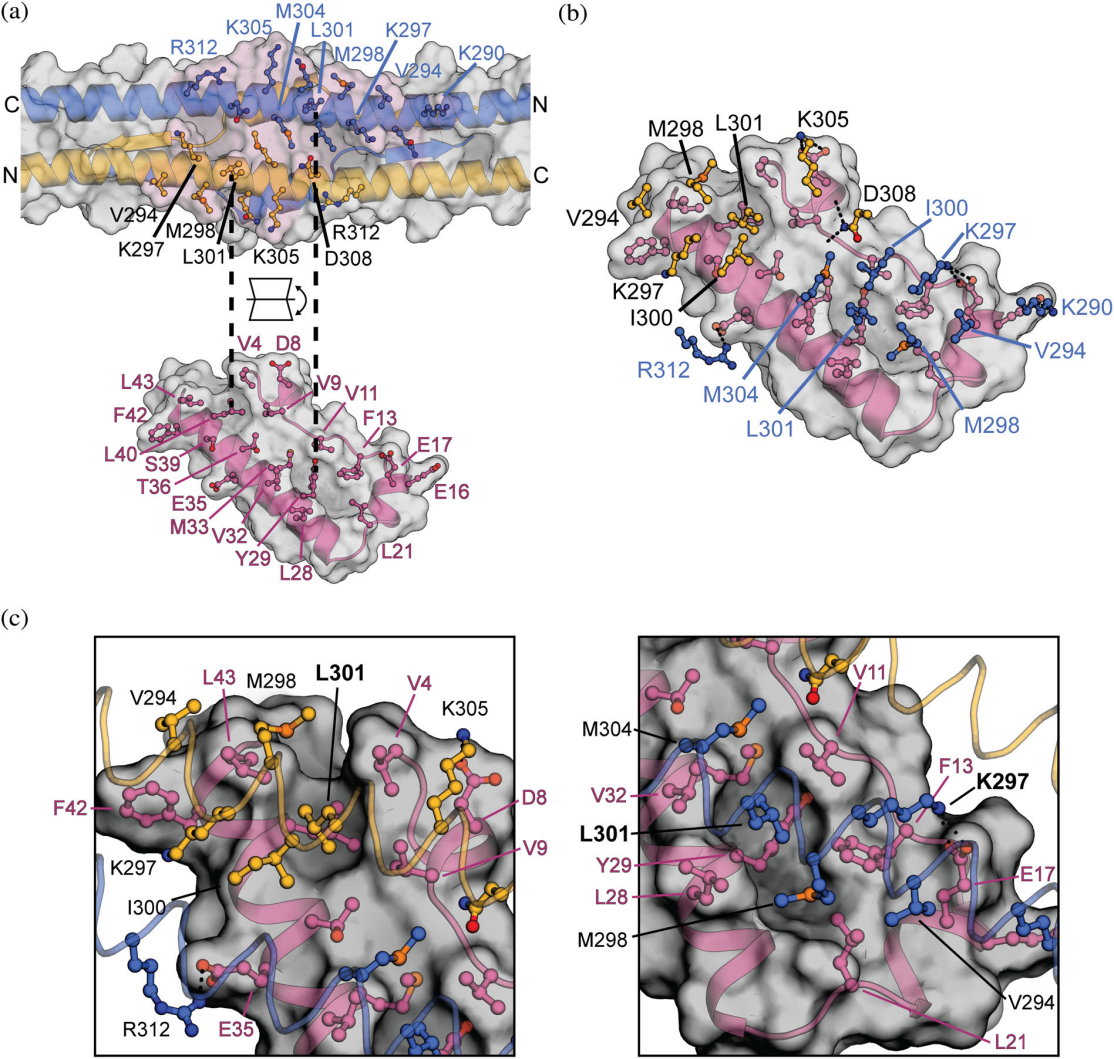
The KRAB interaction centers on L301 from each monomer of Trim28. (a) A cartoon and surface representation of the modeled complex between the Trim28 RBBC and the ZFP809 KRAB domain. An open book representation showing the binding interface with Trim28 chain A/B (blue/gold) and the KRAB domain (magenta). (b) Expanded view of the KRAB binding surface with Trim28 residues shown as ball‐and‐stick representation with potential hydrogen bonds or salt bridge interactions as dashed lines. (c) Expanded view of L301 from each Trim28 monomer located within hydrophobic pockets on the KRAB domain surface

For model interpretation, amino acids with a pLDDT confidence estimate of less than 50 were removed from the model. These residues included the domain linkers from Trim28 and the non‐conserved residues 64–74 of the ZFP809 KRAB domain. The RING and B1 Bbox domains were also removed because they do not contribute to the interaction with the KRAB domain. Our model of the Trim28‐ZFP809 complex reveals an interface with the KRAB domain binding asymmetrically across the two‐fold symmetry axis of the coiled‐coil region of the Trim28 homodimer and the interface buries a surface area of ~1,040 Å^2^ from each molecule. The binding interface with Trim28 spans residues Lys290—Arg312 of chain A, and Val294—Asn308 for chain B (Figure 4). The interface on Trim28 is mediated by two clusters of exposed hydrophobic residues formed by Val294, Met298, Ile300, Leu301, and Met304 on each chain of the Trim28 homodimer. Hydrophobic residues Val294, Met298, Ile300, and Leu301 have previously been identified to contribute in the interaction with KRAB domains.^27^, ^28^

The interaction with the ZFP809 KRAB domain centers on Leu301 from each chain of the Trim28 homodimer, binding two hydrophobic clusters spanning the residue range of Val4—Leu43. Leu301 from chain A binds into the hydrophobic cavity formed by cluster 1 consisting of residues Val11, Phe13, Leu21, Leu28, Tyr29, Val32, and Met33. Moreover, Leu301 from chain B binds into the hydrophobic cavity formed by cluster 2 consisting of residues Val4, Val9, Leu40, Phe42, and Leu43. The complex interface is further stabilized by the surrounding electrostatic interactions. Positively charged residues Lys290, Lys297, and Arg312 of chain A and Lys305 of chain B are modeled in positions consistent with forming salt bridge interactions with highly conserved charged residues Glu16, Glu17, Glu35, and Asp8, respectively. Additionally, chain B Asp308 is modeled in a position consistent with forming hydrogen bond interactions with the backbone oxygens of Asp8 and Val9. In summary, the asymmetry in the interaction is accommodated by the two hydrophobic clusters formed by the KRAB domain that center on Leu301 of each monomer of Trim28.

**FIGURE 5:**
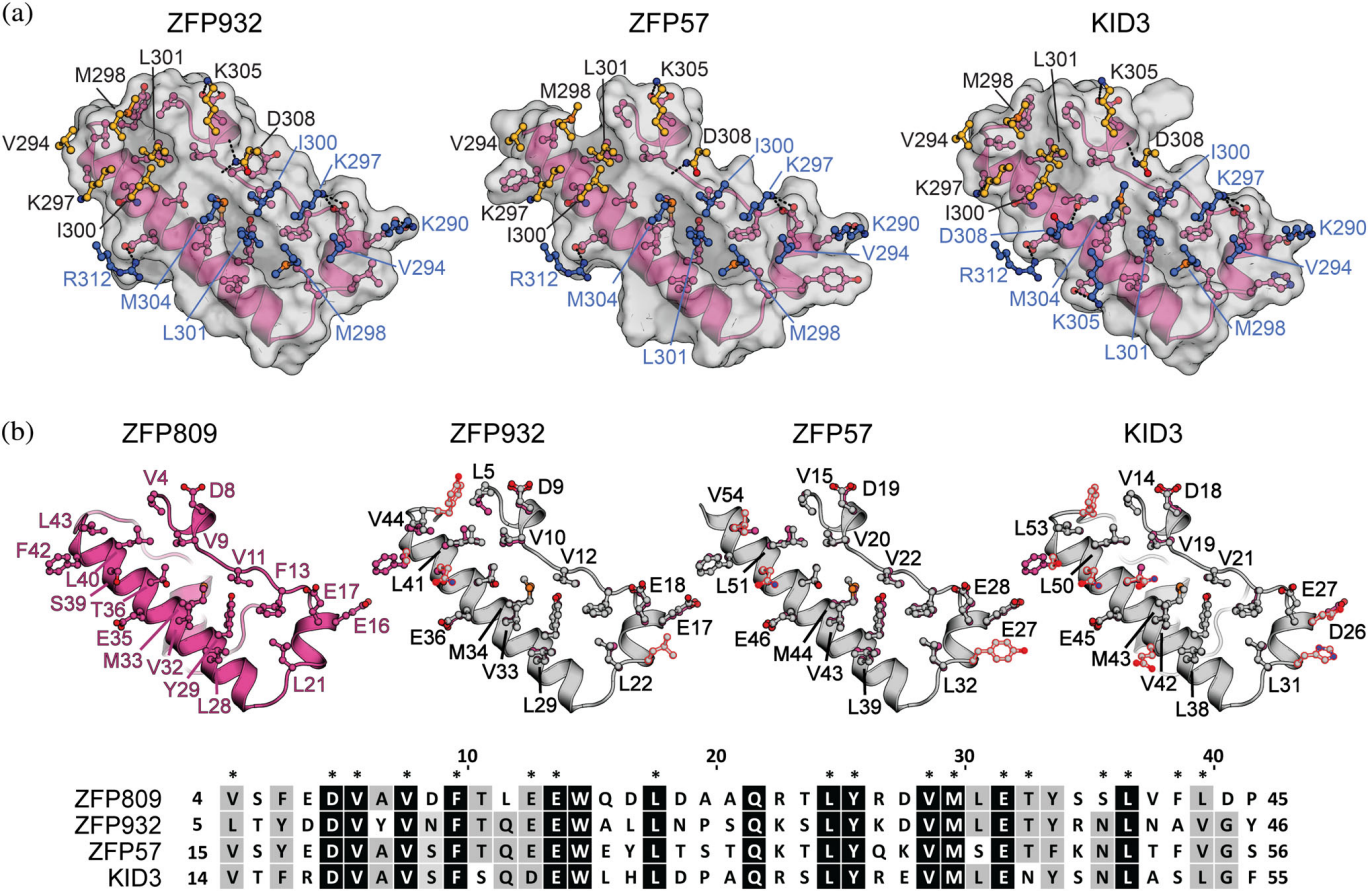
AF2 modeled Trim28‐KRAB complexes share a uniform binding site. (a) Fold outs of the complex models of ZFP932, ZFP57, and KID3 (magenta) with Trim28 chain A/B (blue/gold) residues shown as ball‐and‐sticks representation. (b) Cartoon representations of the modeled KRAB domains with interfacing residues shown as ball‐and‐sticks representation. Structural alignment ZFP809 (magenta) with the KRAB domains of ZFP932, ZFP57, and KID3 (gray). Differences are highlights with a red outline

To assess the consistency in the AF2 modeling we repeated the Trim28‐KRAB complex modeling with the KRAB domains from ZFP932, ZFP57, and KID3. All predicted models were consistent, reproducing the anti‐parallel Trim28 dimer and expected domain organization in all cases. The modeled KRAB domains are all located at the center of the coiled‐coil region in an essentially identical orientation and binding mode, indicating a uniform binding site shared by family members (Figure 5a). The KRAB domains are highly conserved and modeled with near identical sidechain conformations (Figure 5b). ZFP809 and KID3 both have KRAB‐B subdomains that do not participate in the interaction with Trim28 in our models. Only minor differences are observed in the interactions between the 4 KRAB domains and Trim28 that include additional polar and nonpolar contacts from differences in the non‐conserved residues of the KRAB domains. For example, KID3 is modeled with an additional salt bridge interaction with chain A Lys305 to Glu41, and hydrogen bond interactions of chain A Asp308 to Asn46, and chain B Lys297 to Ser52. The ZFP932 model has additional nonpolar contacts through Leu21 and Tyr46, and ZFP57 has additional nonpolar contacts through Tyr31. In summary, KRAB domains bind asymmetrically at the center of the coiled‐coil region and centers on the hydrophobic interactions with Leu301.

### 2.3 Validation of modeled interactions

**FIGURE 6:**
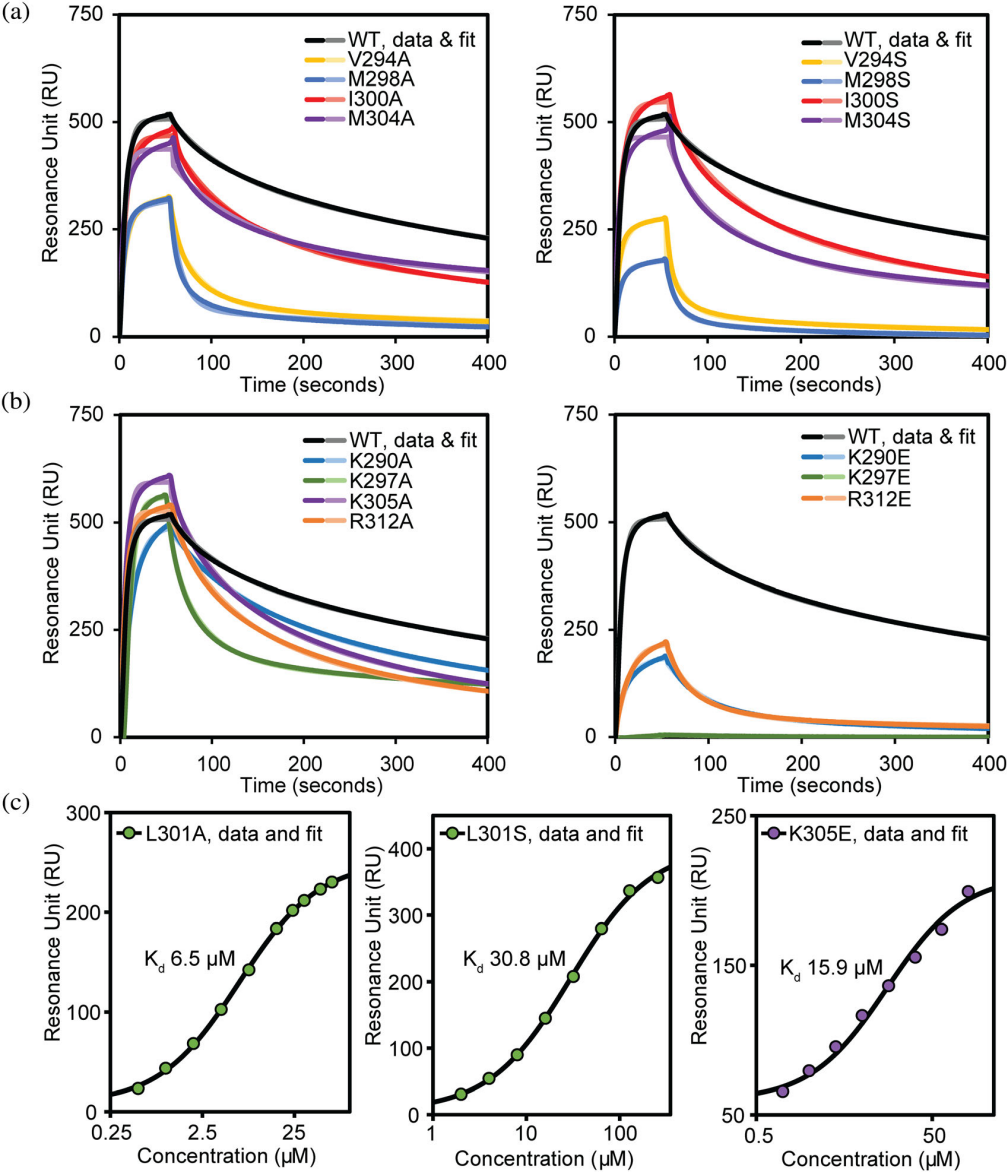
Site‐directed mutagenesis confirms KRAB binding the center of Trim28 coiled‐coil. SPR analysis of Trim28 coiled‐coil mutants over the surface of a CAP sensor chip immobilized with biotinylated Krab domain of ZFP932. (a) Trim28 hydrophobic residues are mutated to alanine and serine (b) charged residues mutated to alanine and glutamate. (c) Equilibrium binding analysis of weak mutants L301A/S and K305E

To validate the complex interface in our structural models we generated site‐directed mutants of Trim28 targeting residues that interact with the KRAB domain, and subsequently measured the binding strength to the KRAB domain of ZFP932 by SPR (Figure 6 and Table 2). Nine Trim28 residues were individually mutated to the small neutral alanine to disrupt hydrophobic and polar interactions (K290A, V294A, K297A, M298A, I300A, L301A, M304A, K305A, and R312A). The five hydrophobic residues were also individually mutated to serine to disrupt the hydrophobic core (V294S, M298S, I300S, L301S, and M304S), and the four charged residues individually mutated to glutamate to disrupt the salt bridge interactions (K290E, K297E, K305E, and R312E).

**TABLE 2:** Summary of residues targeted for mutation in Trim28 coiled‐coil and the affinity to ZFP932 KRAB domain

Mutant name	Dissociation constant (nM)	Fold‐decrease	Mutant name	Dissociation constant (nM)	Fold‐decrease
Wild type	14.6 ± 1.1				
K290A	37.3 ± 0.8	3	K290E	132.3 ± 16.0	9
V294A	113.3 ± 10.9	8	V294S	319.0 ± 28.6	22
K297A	54.1 ± 9.0	4	K297E	Binding‐deficient	—
M298A	323.7 ± 27.1	22	M298S	670.6 ± 61.3	46
I300A	26.3 ± 2.3	2	I300S	35.8 ± 0.9	2
L301A	6.5 ± 0.5 μM	445	L301S	30.8 ± 1.0 μM	2,112
M304A	24.2 ± 1.0	2	M304S	20.3 ± 2.2	2
K305A	31.1 ± 3.0	2	K305E	15.9 ± 0.4 μM	1,071
R312A	27.8 ± 2.1	2	R312E	212.1 ± 27.1	15

*Note*: Residues at the center of the MuTrim28 coiled‐coil region were mutated to alanine and serine or glutamate. The binding strength of the Trim28 coiled‐coil mutants to the ZFP932 KRAB domain were assessed by SPR. The data are presented as the average of three equivalent runs plus minus the standard deviation. The affinities are presented in nM, unless otherwise specified.

The KRAB domain was expressed with an N‐terminal Avitag resulting in a monobiotinylated construct that could be immobilized onto the surface of a biacore Sensor Chip CAP. Data were analyzed by either equilibrium or kinetics analysis. Analysis of the K297E mutant reveals this is the most disruptive to binding and completely abolishes the interaction with the KRAB domain. In our AlphaFold models Lys297 is buried in the interface and on chain A is modeled in a position consistent with forming a salt bridge interaction with the conserved Glu18 of ZFP932, thus mutation of Lys297 to glutamate would likely result in a charge repulsion. Substituting Lys297 for alanine resulted in a minor disruption in binding with an affinity of 54.1 ± 9.0 nM, a four‐fold decrease in binding strength. Mutations in L301 are the second most disruptive to the interaction with ZFP932 with the mutation to alanine resulting in a 445‐fold decrease in affinity with a K_d_ of 6.5 ± 0.5 μM, and mutation to serine resulting in a K_d_ of 30.8 μM, a 2,112‐fold decrease in affinity. This is consistent with L301 on each chain of the Trim28 homodimer binding into hydrophobic cavities in the Trim28‐KRAB complex models, where alanine would be too small to occupy the cavity and serine would be unfavorable.

Mutations in the hydrophobic residues V294A and M298A also resulted in moderate to severe reduction in binding strength with affinities of 113.3 ± 10.9 nM and 323.7 ± 27.1 nM, respectively. Binding is further diminished for V294S and M298S with affinities of 319 ± 28.6 nM and 670.6 ± 61.3 nM, respectively, and is consistent with these residues being buried in the central hydrophobic groove of the KRAB domain in our models. Mutants K290A, I300A, M304A, K305A, and R312A all resulted in a modest reduction in binding strength with a 6–23‐fold decrease in affinity. Mutating Ile300 and Met304 to serine further disrupted the interaction to ZFP932, but have a smaller impact on binding than the other hydrophobic resides Val294, Met298, and Leu301. R312E resulted in a modest reduction in the binding strength of 212.1 ± 27.1 nM, likely due to only Arg312 from chain A participating in the interaction, Arg312 from Chain B is not involved. Conversely, Lys305 from each chain of the homodimer is buried at the center of the binding interface and the K305E mutation results in a severe reduction in binding with an affinity of 15.9 ± 0.4 μM, a 1,071‐fold decrease in binding. SEC‐MALLS analysis of the K297E and L301S mutants showed the overall fold of the mutants was not disrupted as all share identical elution volumes and MW with the wild‐type Trim28 (Figure S2). In conclusion, our mutagenesis data validate our model that the KRAB domain binds to the central region of Trim28 coiled‐coil. The interaction involves the key hydrophobic and charged residues, with Leu301 and Lys297 being the most important residues in binding.

## 3 DISCUSSION

The interaction between Trim28 and the KRAB‐ZFPs is responsible for targeting Trim28 to a specific locus in the genome and is central to silencing. The KRAB‐ZFP locates a single site within the genome and Trim28 recruits the machinery to initiate silencing. Previous *in vitro* investigations of Trim28 binding to the KRAB domain of KRAB‐ZFPs determined the KRAB domain binds at the center of the coiled‐coil domain,^27^, ^28^ and mutagenesis of surface exposed residues V294 and K297 located at the center of the coiled‐coil moderately reduce or abrogate binding. Our Biacore analysis of the coiled‐coil turn mutants demonstrates that residues located on Turns 1–6 at the center of the coiled‐coil are involved in the interaction expanding the binding interface compared to previous studies.^27^, ^28^

Alphafold2 has been shown to predict structures for single protein chains with a high degree of confidence, including folds that are normally stabilized by multimeric interactions. This obviously leads to the investigation of using AF2 to predict protein–protein interfaces. Indeed, the physical properties that underpin protein folding are not different than those that govern protein–protein interactions. The development of AF‐multimer trained on multimeric inputs and recent work on AF2Complex is seeking to make complex modeling more accurate and routine.^31^, ^33^ While still in its infancy for predicting protein complexes, the interaction between Trim28 and the KRAB domain is an ideal model to examine its efficacy. This interaction is high‐affinity and required for development with Trim28 knock‐out mouse mutants being embryonic lethal at day 5.5.^34^ This suggests that the interaction will carry sufficient co‐evolutionary information required for the complex interface modeling to be accurate.

Our modeling of Trim28 in complex with KRAB domains produced high accuracy predictions consistent with known structures Trim28 and the KRAB domain in isolation.^15^, ^27^ Additionally, placement of the KRAB domain at the center of the coiled‐coil is consistent with our mutagenesis data and low‐resolution models of the complex obtained from SAXS data.^28^ To probe the consistency in AF2 modeling we modeled the complex with the KRAB domains from ZFP809, ZFP932, ZFP57, and KID3 showing a uniform binding site and identical binding mode across all models. These models demonstrate how the 2:1 binding stoichiometry of a Trim28 dimer binding to a single KRAB domain is accommodated. In our models L301 from each Trim28 monomer acts as a pin that locates within a hydrophobic pocket on the KRAB domain surface. The KRAB domain binds directly across the two‐fold symmetry axis of the Trim28 dimer occluding a second possible KRAB domain binding site. The interface is further stabilized by additional hydrophobic and polar contacts. To validate and probe this model, we determined the ZFP932 KRAB domains binding affinity for the Trim28 mutant L301A (K_d_ = 6.5 ± 0.5 μM) to be 445‐fold weaker than wildtype, and L301S (K_d_ = 30.8 ± 1.0 μM) to further diminished binding by 2,112‐fold. This mutagenesis confirms the importance of L301 with mutations to surrounding residues also resulting in a reduction in binding affinity but to a lesser degree.

The asymmetric interface formed by the Trim28:KRAB complex is extremely tight in fitting with its biological role, the KRAB domain must recruit Trim28 to a single site within the genome. The two pseudo symmetric hydrophobic pockets on the KRAB domain allows it to bind in either orientation with essentially identical interactions. Comparison with the structure of the Trim28—SMARCAD1 Cue1 complex shows no overlap in the binding interface allowing both interactions to occur simultaneously. This is consistent with the Trim28 dimer functioning as a scaffold tethering multiple epigenetic regulators to KRAB‐ZFP binding sites.

Consistent with the models presented, mutation of conserved amino acids within the KRAB‐A domain have previously been shown to abrogate Trim28‐mediated transcriptional repression.^7^, ^35^, ^36^ These studies identified and described highly conserved sequence motifs from ZFP10 and ZFP748 that are shared by the KRAB‐ZFP protein family. These sequences, including the “DV,” “EEW,” and “MLE” motifs, disrupt the interaction with Trim28 and transcriptional repression when mutated (Figure S3). Comparing the conserved KRAB‐A sequences to homologous residues from ZFP809 in our modeled complex shows they are located within in the binding interface and contribute to hydrophobic and polar contacts. The ZFP809 residues D8, E17, and E35 from the “DV,” “EEW,” and “MLE” motifs form salt bridge interactions with Trim28 residues K297, K305, and R312, and the KRAB residues V9 and M33 form part of the hydrophobic pocket that interacts with L301. Additional hydrophobic resides V11, F13, L21, L28, and V32 form part of the hydrophobic core contacting L301 from Trim28 contributing to the interaction. These functional studies are congruent with our site‐directed mutagenesis of Trim28 targeting the interfacing hydrophobic residues V294, M298, and L301 that results in the reduction in the binding affinity. The binding interface described in our Trim28‐KRAB complex models provide the molecular understanding for how these mutations exhibit their effect on binding and the subsequent phenotype.

The KRAB‐B box has previously been shown to enhance the repression activity of the KRAB‐A box.^8^, ^10^ To probe the role of the KRAB‐B box in the interaction with Trim28 we modeled the complex with the KRAB domains from ZFP809 and KID3 that are members of the KRAB A + B class containing a KRAB‐A box and KRAB‐B box, and ZFP932 and ZFP57 that are members of the KRAB A class containing only a KRAB‐A box. The KRAB‐B box does not participate in the binding interface in our models and is consistent with the observation that the KRAB‐B box does not result in a higher affinity for Trim28.^28^ Therefore, the KRAB‐B box does not potentiate repression by contributing to the binding interface, and the mechanism for the auxiliary effects on silencing remain unclear.

Our study provides insights into the initial events during Trim28‐mediated transcription silencing. The affinity of Trim28 for the KRAB domain is low nanomolar ranging from 2 to 200 nM.^28^ The stoichiometry and affinity are congruent with the purpose of the interaction whereby a KRAB‐ZFP locates a single site in the genome that is targeted for silencing. The interaction with dimeric Trim28 then recruits the epigenetic machinery for robust transcriptional repression. Our models provide a detailed understanding of the specific interactions between the KRAB domain and the Trim28 coiled‐coil and how this interaction may be regulated during silencing events.

## 4 METHODS

### 4.1 Cloning

DNA encoding murine Trim28 (1–834) and ZFP932_KRAB (5–76) was codon optimized and synthesized (GeneArt). The coding sequences for T28_RBCC (58–418) and ZFP932 (5–76) were amplified by PCR. These constructs were cloned into pET‐49b(+)‐MBP (N‐terminal MBP‐His‐tag, made in house by replacing the *g*lutathione *S*‐*t*ransferase [GST] tag in pET49b with an *m*altose *b*inding *p*rotein [MBP] tag) by a ligation independent cloning (LIC) method.^37^ The resulting constructs contain an N‐terminal purification tag followed by a 3C protease cleavage site to allow tag removal post affinity purification. The coiled‐coil variants were generated by whole plasmid PCR using the wild‐type plasmid as template and verified by DNA sequencing. To generate biotinylated the KRAB domain, the DNA sequence encoding a specific BirA biotinylation tag GLNDIFEAQKIEWHE (AviTag) was codon optimized and inserted downstream of the 3C protease cleavage site upstream of protein start site using whole plasmid PCR. All plasmid constructs were verified by DNA sequencing.

### 4.2 Protein expression and purification

All Trim28 constructs were expressed in *LOBSTR* (DE3) cells^38^ grown in LB media supplemented with 100 μM ZnCl. Expression was induced by the addition of 1 mM isopropyl β‐_D_‐1‐thiogalactopyranoside (IPTG) to log phase cultures prior to subsequent growth overnight at 18°C. Cells were lysed by cell disruption in 20 mM Tris–HCl (pH 8.0), 200 mM NaCl, 0.5 mM Tris(2‐carboxyethyl)phosphine (TCEP), 10% (v/v) glycerol, 0.1% (v/v) Triton X‐100. The MBP‐His‐tagged Trim28 constructs were purified using amylose affinity chromatography. The N‐terminal purification tags were removed by cleavage with 3C‐protease prior to further purification by size exclusion chromatography (SEC). The AviTagged ZFP932 KRAB domain was co‐expressed with pBirA in *E. coli* BL21 (DE3) LOBSTR grown in LB at 37°C to approximately 0.5 OD_600_. Cells were then induced with 1 mM IPTG and supplemented with 20 μM D‐biotin (Sigma) and grown overnight at 18°C. The MBP‐His‐tagged ZFP932_KRAB (5–76) was then purified as described above.

### 4.3 SEC‐MALLS

Size‐exclusion chromatography coupled to multi‐angle laser light scattering (SEC‐MALLS) was used to determine the solution molecular weight and assess protein heterogeneity. Samples (100 μl) were applied to either a Superdex S200 Increase 10/300GL column equilibrated in 10 mM Tris/HCl pH 7.8, 150 mM NaCl, 0.1 mM TCEP mounted on a Dionex HPLC with a PSS SLD7000 7‐angle MALLS detector and Shodex RI‐101 differential refractive index detector. The weight average molecular weight was determined using PSS winGPC Unichrom software.

### 4.4 Surface plasmon resonance

SPR was undertaken using a Sensor Chip CAP in a Biacore X100 instrument. CAPture reagent (GE) was loaded onto both the reference and sample surfaces at a flow rate of 5 μl/min for 120 s, achieving a resonance unit (RU) of ~2,500. Monobiotinylated KRAB domain was then loaded onto the sample surface at 10 μl/min achieving ~100 RU. Exactly, 1 μM of RBCC_R184D or RBCC coiled‐coil mutants were injected over the reference and sample surfaces for 60 s, followed by a 600 s wash with SPR buffer (10 mM TRIS/HCl, pH 8.0, 300 mM NaCl, 0.1 mM TCEP, 0.005% tween 20). K_d_ was determined by fitting a two‐state reaction model using BiacoreX100 Evaluation Software version 2.0.1. For equilibrium analysis, a protein concentration series was prepared by serial diluting proteins with SPR buffer. Each concentration (from low to high) was injected over the reference and sample surfaces for 60 s, followed by a 600 s wash with SPR buffer. The data was fitted using BiacoreX100 Evaluation Software version 2.0.1.

### 4.5 Alphafold2

Models were produced using AlphaFold2_advanced on Google Colab GPUs. The input were sequences of Trim28 RBCC (residues 58–418) and KRAB domains with the stoichiometry set to 2:1 Trim28:KRAB. No templates were included in the modeling and the models were relaxed using Amber‐Relax as part of the AF2 modeling process. Five models were produced for each complex and the rank1 model that has the highest overall confidence was chosen for analysis. No differences were observed in the Trim28:KRAB interface between the 5 models. Residues with pLDDT less than 50 and the B1 Bbox domains were removed prior to analysis.

## AUTHOR CONTRIBUTIONS

**Jamie R. H. Taka:** Conceptualization (supporting); investigation (equal); writing – original draft (equal); writing – review and editing (equal). **Yunyuan Sun:** Investigation (supporting); writing – review and editing (supporting). **David C. Goldstone:** Conceptualization (equal); investigation (equal); supervision (equal); writing – original draft (equal); writing – review and editing (equal).

## CONFLICT OF INTEREST

The authors declare no competing financial interests.

## Supporting information


**Figure S1**. Structural comparison between the modeled Trim28‐KRAB complexes indicates a uniform binding site. Predicted aligned error plots for the four models of the Trim28‐KRAB complexes with Trim28 consisting of chain A and B, and the KRAB domains consisting of chain C. The plots indicate a high confidence in the relative position of residues within the domains of the Trim28 RBCC. There is high confidence in the relative position of the KRAB domains to the coiled‐coil regions of each chain of Trim28.
**Figure S2**. SEC‐MALLS analysis of the Trim28 RBCC coiled‐coil mutants. Protein elution profile is shown by differential refractive index traces (solid lines) and M_W_ is represented by open circles. (a–c) Mutation of coiled‐coil residues to alanine and change of function mutations show a consistent elution profile and M_w_ as wildtype Trim28. (d) Mutation of K305E has weak concentration dependent self‐association.
**Figure S3**. Mutation of conserved residues in the KRAB‐A box disrupts the interaction with Trim28 and transcriptional repression. The KRAB‐A box is highly conserved and responsible for the interaction with Trim28. Residues involved in the Trim28‐KRAB binding interface with from our AF2 models are marked by an asterisk. GST‐pulldown binding assay shows mutations of conserved KRAB‐A box residues disrupt the interaction with the Trim28 RBCC (Peng et al., 2009) and are congruent with a disruption of the binding interface identified from our structural models. GAL4‐KRAB‐A box mutations also disrupt the transcriptional repression activity of KZFPs (Lorenz et al., 2022; Margolin et al., 1994; Peng et al., 2009). Exchanging the PRDM9 residues for amino acids occurring at the respective positions in ZFP10 confers repression activity, whereas wildtype PRDM9 is unable to repress transcription. (B) ZFP809 KRAB domain model showing location of conserved “D_8_V_9_,” “E_16_E_17_W_18_,” and “M_33_L_34_E_35_” motifs involved in the interaction with Trim28
