# Supplementary material for: Mapping the interaction between Trim28 and the KRAB domain at the center of Trim28 silencing of endogenous retroviruses
Source: Protein Sci. 2022 Sep 21;31(10):e4436. doi: 10.1002/pro.4436 (PMC9601868; doi:10.1002/pro.4436)
Supplement: Supplementary file 1 — Figure S1. Structural comparison between the modeled Trim28‐KRAB complexes indicates a uniform binding site. Predicted aligned error plots for the four models of the Trim28‐KRAB complexes with Trim28 consisting of chain A and B, and the KRAB domains consisting of chain C. The plots indicate a high confidence in the relative position of residues within the domains of the Trim28 RBCC. There is high confidence in the relative position of the KRAB domains to the coiled‐coil regions of each chain of Trim28. Figure S2. SEC‐MALLS analysis of the Trim28 RBCC coiled‐coil mutants. Protein elution profile is shown by differential refractive index traces (solid lines) and MW is represented by open circles. (a–c) Mutation of coiled‐coil residues to alanine and change of function mutations show a consistent elution profile and Mw as wildtype Trim28. (d) Mutation of K305E has weak concentration dependent self‐association. Figure S3. Mutation of conserved residues in the KRAB‐A box disrupts the interaction with Trim28 and transcriptional repression. The KRAB‐A box is highly conserved and responsible for the interaction with Trim28. Residues involved in the Trim28‐KRAB binding interface with from our AF2 models are marked by an asterisk. GST‐pulldown binding assay shows mutations of conserved KRAB‐A box residues disrupt the interaction with the Trim28 RBCC (Peng et al., 2009) and are congruent with a disruption of the binding interface identified from our structural models. GAL4‐KRAB‐A box mutations also disrupt the transcriptional repression activity of KZFPs (Lorenz et al., 2022; Margolin et al., 1994; Peng et al., 2009). Exchanging the PRDM9 residues for amino acids occurring at the respective positions in ZFP10 confers repression activity, whereas wildtype PRDM9 is unable to repress transcription. (B) ZFP809 KRAB domain model showing location of conserved “D8V9,” “E16E17W18,” and “M33L34E35” motifs involved in the interaction with Trim28 [file PRO-31-e4436-s001.docx]

Mapping the interaction between Trim28 and the KRAB domain at the centre of Trim28 silencing of endogenous retroviruses.

Jamie R. H. Taka^1^, Yunyuan Sun^1^, David C. Goldstone^1,2,*^

*^1^School of Biological Sciences, University of Auckland, Auckland, New Zealand*

*^2^Associate Investigator, Maurice Wilkins Centre for Molecular Biodiscovery*

*To whom correspondence should be addressed

Telephone: +64 9 923 4607

E-mail: [d.goldstone@auckland.ac.nz](mailto:d.goldstone@auckland.ac.nz)

Runnnig title: Mapping the Trim28-KRAB interaction with Alphafold2

Manuscript Pages:

Tables:

Figures:

Supplementary Material (Pages, contents, filenames):

Abbreviations: KRAB-ZFPs - KRAB-containing zinc finger proteins

***SUPPLEMENTARY MATERIAL***


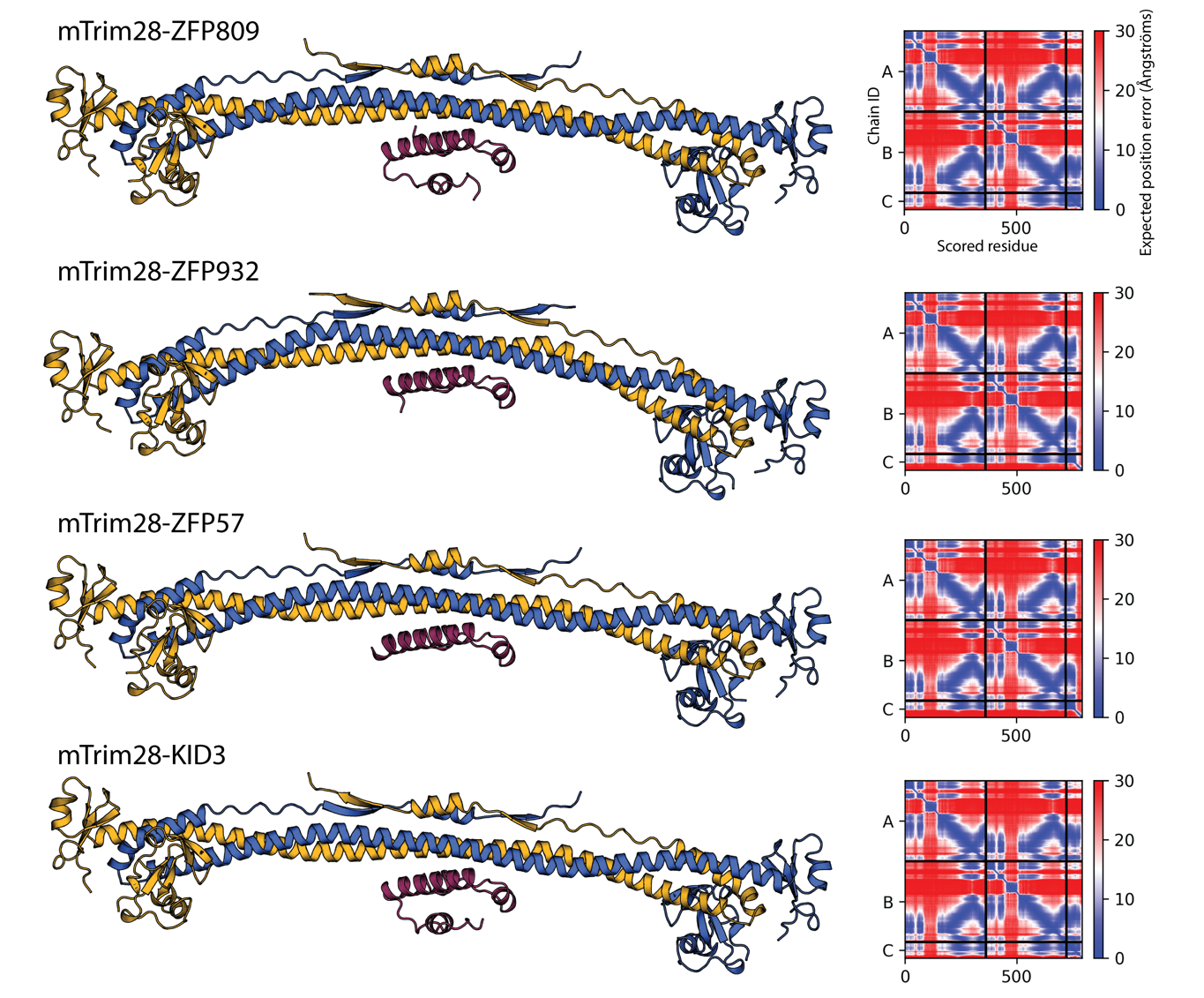


# **Figure S1. Structural comparison between the modelled Trim28-KRAB complexes indicates a uniform binding site.** Predicted aligned error plots for the four models of the Trim28-KRAB complexes with Trim28 consisting of chain A and B, and the KRAB domains consisting of chain C. The plots indicate a high confidence in the relative position of residues within the domains of the Trim28 RBCC. There is high confidence in the relative position of the KRAB domains to the coiled-coil regions of each chain of Trim28.


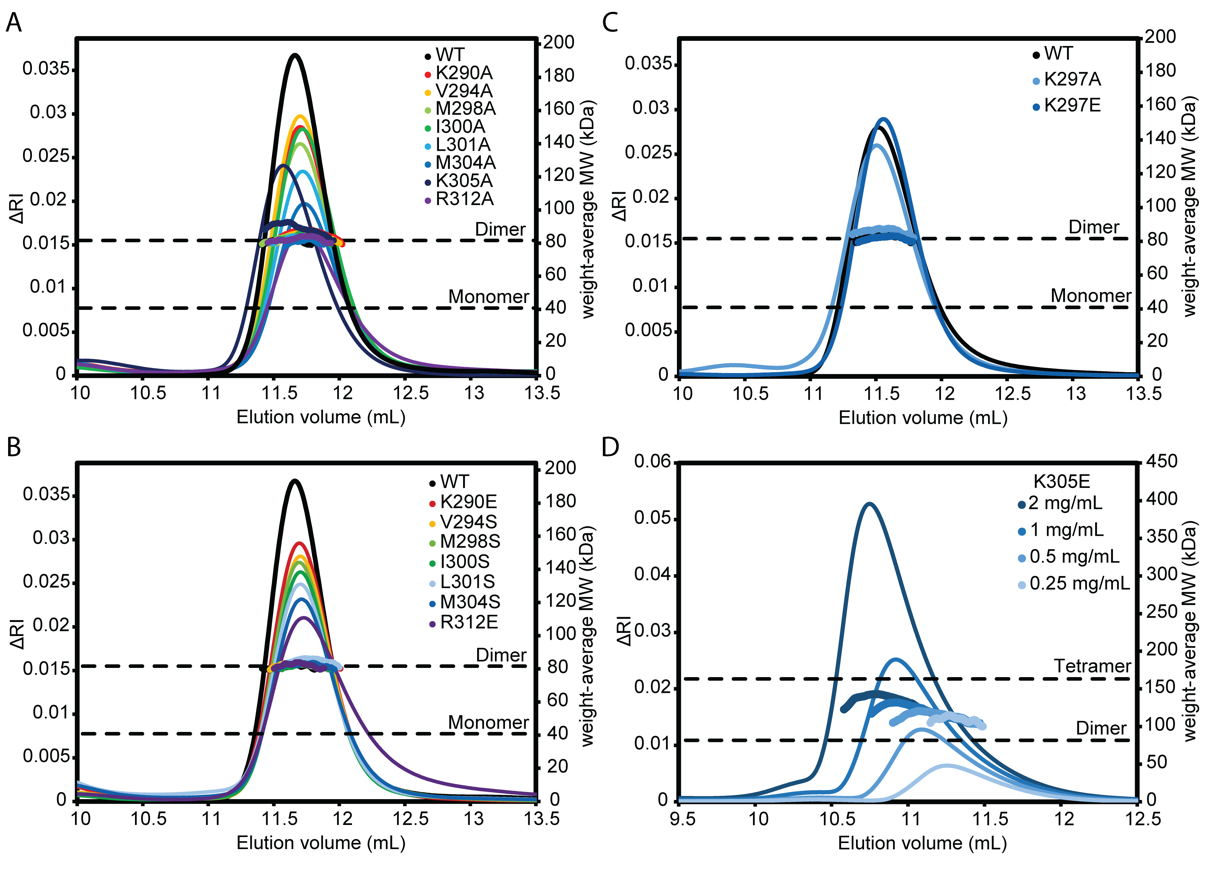


# **Figure S2. SEC-MALLS analysis of the Trim28 RBCC coiled-coil mutants.** Protein elution profile is shown by differential refractive index traces (solid lines) and M_W_ is represented by open circles. (A-C) Mutation of coiled-coil residues to alanine and change of function mutations show a consistent elution profile and M_w_ as wildtype Trim28. (D) Mutation of K305E has weak concentration dependent self-association.


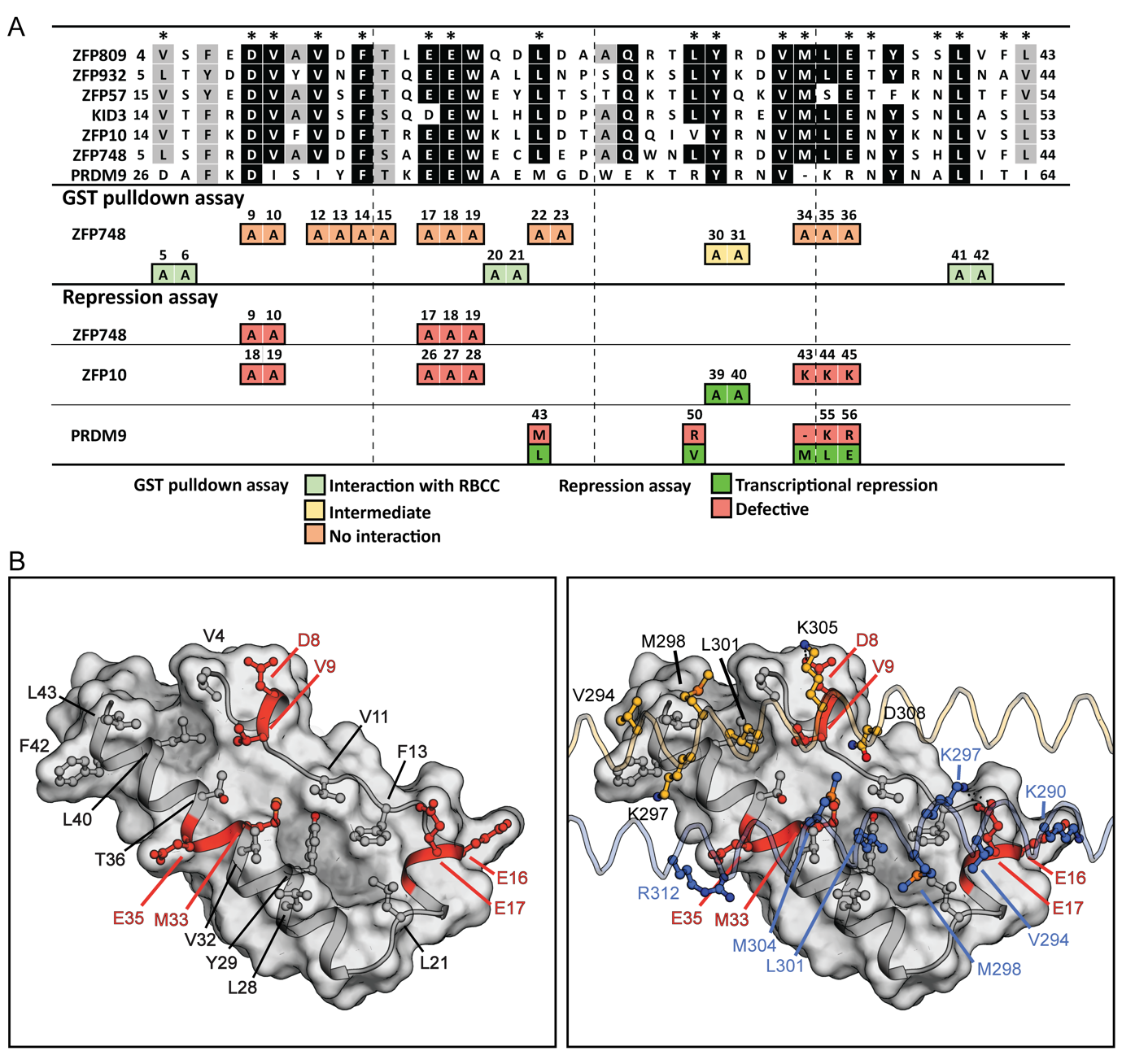


# **Figure S3. Mutation of conserved residues in the KRAB-A box disrupts the interaction with Trim28 and transcriptional repression.** The KRAB-A box is highly conserved and responsible for the interaction with Trim28. Residues involved in the Trim28-KRAB binding interface with from our AF2 models are marked by an asterisk. GST-pulldown binding assay shows mutations of conserved KRAB-A box residues disrupt the interaction with the Trim28 RBCC (Peng et al., 2009) and are congruent with a disruption of the binding interface identified from our structural models. GAL4-KRAB-A box mutations also disrupt the transcriptional repression activity of KZFPs (Lorenz et al., 2022; Margolin et al., 1994; Peng et al., 2009). Exchanging the PRDM9 residues for amino acids occurring at the respective positions in ZFP10 confers repression activity, whereas wildtype PRDM9 is unable to repress transcription. (B) ZFP809 KRAB domain model showing location of conserved “D_8_V_9_” “E_16_E_17_W_18_” and “M_33_L_34_E_35_” motifs involved in the interaction with Trim28
